# Sucrose and ABA regulate starch biosynthesis in maize through a novel transcription factor, *ZmEREB156*

**DOI:** 10.1038/srep27590

**Published:** 2016-06-10

**Authors:** Huanhuan Huang, Sidi Xie, Qianlin Xiao, Bin Wei, Lanjie Zheng, Yongbin Wang, Yao Cao, Xiangge Zhang, Tiandan Long, Yangping Li, Yufeng Hu, Guowu Yu, Hanmei Liu, Yinghong Liu, Zhi Huang, Junjie Zhang, Yubi Huang

**Affiliations:** 1 College of Agronomy, Sichuan Agricultural University, Chengdu 611130, Sichuan, China; 2 College of Life Science, Sichuan Agricultural University, Chengdu 611130, Sichuan, China; 3 Maize Research Institute, Sichuan Agricultural University, Chengdu 611130, Sichuan, China; 4 College of Horticulture, Sichuan Agricultural University, Chengdu 611130, Sichuan, China

## Abstract

Sucrose is not only the carbon source for starch synthesis, but also a signal molecule. Alone or in coordination with ABA, it can regulate the expression of genes involved in starch synthesis. To investigate the molecular mechanisms underlying this effect, maize endosperms were collected from *Zea mays* L. B73 inbred line 10 d after pollination and treated with sucrose, ABA, or sucrose plus ABA at 28 °C in the dark for 24 h. RNA-sequence analysis of the maize endosperm transcriptome revealed 47 candidate transcription factors among the differentially expressed genes. We therefore speculate that starch synthetic gene expression is regulated by transcription factors induced by the combination of sucrose and ABA. *ZmEREB156*, a candidate transcription factor, is induced by sucrose plus ABA and is involved in starch biosynthesis. The *ZmEREB156*-GFP-fused protein was localized in the nuclei of onion epidermal cells, and *ZmEREB156* protein possessed strong transcriptional activation activity. Promoter activity of the starch-related genes *Zmsh*2 and *ZmSSIIIa* increased after overexpression of *ZmEREB156* in maize endosperm. *ZmEREB156* could bind to the *ZmSSIIIa* promoter but not the *Zmsh2* promoter in a yeast one-hybrid system. Thus, *ZmEREB156* positively modulates starch biosynthetic gene *ZmSSIIIa* via the synergistic effect of sucrose and ABA.

Starches produced by higher plants function as seed storage reserve carbohydrates and are the most important dietary source of energy for humans, representing a major proportion of daily caloric intake^1^. The storage starches produced in maize endosperm amyloplasts account for over 90% of the world market for starch^2^. Starch biosynthesis and accumulation is an important process that not only determines grain yield but also influences grain quality^3^. Starch biosynthesis in the cereal endosperm requires the coordinated activities of several major enzymes, including adenosine 5′ diphosphate-glucose (ADP-Glc) pyrophosphorylase (AGPase), starch synthase (SS), starch branching enzyme (SBE), and starch debranching enzyme (DBE)^4^,^5^. However, the molecular mechanisms that regulate the gene expression of the network of starch synthesis enzymes remain unclear^6^.

Sucrose (Suc) is an important source of energy and carbon skeletons for plant growth and development, but also acts as an important signal that modulates developmental and metabolic processes in the plant life cycle^7^,^8^. It has been reported that sucrose acts as a signalling molecule for genes critical to starch biosynthesis in different species^9^,^10^. Sucrose is the only sugar capable of inducing the expression of the AGPase large subunit genes (*iAGPLI-1* and *ApL3*) in sweet potato and *Arabidopsis thaliana*^11^,^12^. Both starch synthase (GBSSI) and β-amylase genes are also reportedly induced by sucrose in sweet potato^13^,^14^. Kim and Guiltinan showed that expression of the *Sbe1* gene was induced by sucrose and was regulated through *mEmBP-1*, a bZIP transcription activator, in suspension-cultured maize endosperm cells, suggesting a possible regulatory role of the C-box present in the *Sbe1* promoter from −227 to −220^15^. Sun found that the transcription factor *SUSIBA2*, induced by sucrose, belongs to the WRKY protein family and binds to sucrose-responsive elements (SURE) and W-box elements but not to the SP8a element in the *iso1* promoter^16^. These reports confirm the importance of sucrose as a signalling molecule, but the molecular mechanism is not fully understood.

In a previous study, we found that sucrose combined with abscisic acid (ABA) synergistically influenced expression of 15 starch biosynthetic genes in maize endosperm^17^. Few reports indicate that sucrose induces starch biosynthetic gene expression by interacting with ABA signalling pathways^18^,^19^,^20^,^21^. *AtAPL*3 and *OsAPL*3 expression increased in response to exogenous application of sucrose in *Arabidopsis* leaves and cultured rice cells, respectively^20^,^21^. In addition, their expression was further enhanced by co-treatment with ABA. However, the molecular mechanisms by which starch biosynthetic genes in maize endosperm are regulated by interaction with sucrose and ABA remain unclear. In the present study, maize endosperms 10 DAP (days after pollination) were treated with sucrose, ABA, or both, and then analysed by RNA sequencing (RNA-seq). Analysis of these gene sets identified different treatments of gene expression, including hundreds of transcription factor genes. We found that some transcription factor genes were affected synergistically by Sucrose and ABA. We hypothesized that at least one transcription factor gene would be involved in maize endosperm starch synthesis by synergistic effect of sucrose combined with ABA. The results lay a foundation for understanding the underlying mechanisms that control seed yield and quality.

## Results

### RNA sequencing and data analysis

To analyse global gene expression in maize endosperm in response to sucrose or/and ABA signalling, maize endosperms were collected ten DAP and treated with sucrose (Suc), abscisic acid (ABA), or both (Suc + ABA). Mannitol was added to the samples without sugars as an osmotic control. Four cDNA libraries were constructed from total RNAs extracted and analysed sequences on the Illumina HiSeq^TM^ 2000. After quality control, approximately 214 million valid reads and roughly 19.3 Gb of nucleotides were obtained. The generated reads were then aligned to the maize reference gene set based on B73 genome (release 5b.60) by applying the SOAPaligner/SOAP2 programs^22^. Sample data from the four libraries were summarized in Table 1. About 62% of the reads from each sample perfectly matched the gene set, 15–16.8% of the reads mapped to the gene set with no more than five aligned positions, and about 74–78% of the RNA-seq reads mapped to a unique position in the gene set.

Since reference genes have different lengths, the read location on each gene is standardized to a relative position (which is calculated as the ratio between read location on the gene and the gene length), and then the number of reads in each relative position is counted. The reads on the reference genes of our libraries were evenly distributed (Supplementary Figure S1), indicating that the randomness of the reads was satisfactory. As shown in Supplementary Figure S2, the distribution pattern of unique reads over different read abundance categories was similar for all four RNA-seq libraries.

### Statistical analyses of differentially expressed genes (DEGs)

**Figure 1:**
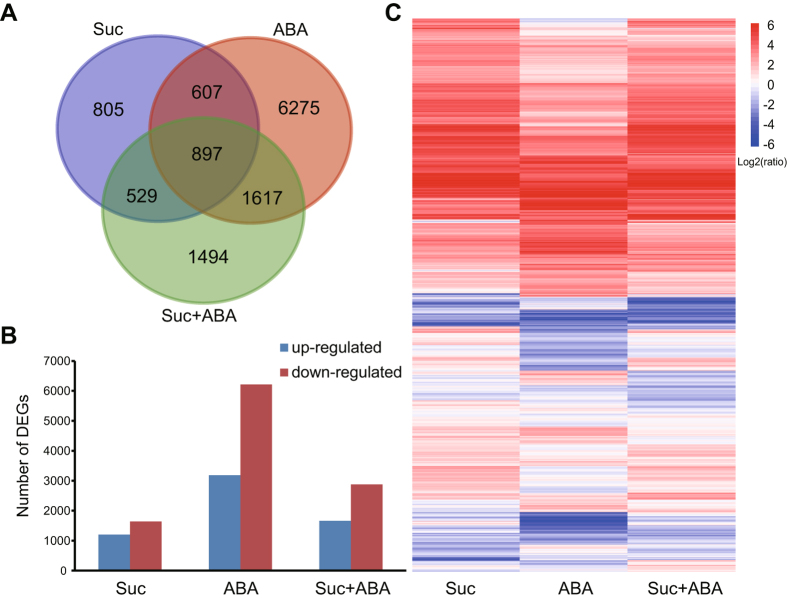
Number of differentially expressed genes (DEGs) after treatment with sucrose, ABA, or both. DEGs are identified by comparison with control (FDR < 0.01, |log_2_ Ratio| ≥ 1). (**A**) Venn diagram of the number of DEGs in three comparisons; (**B**) Number of up- or down-regulated DEGs in each comparison; (**C**) The heatmap of DEGs using hierarchical clustering.

Uniquely mapped reads were used to estimate normalized transcription level as reads per kilobase of transcript per million mapped reads (RPKM). To identify genes displaying significant expression changes during Suc, ABA, or Suc + ABA treatment, DEGs (FDR < 0.01, |log_2_ Ratio| ≥ 1) were analysed by comparing the Suc, ABA, and Suc + ABA libraries with the control library, (i.e. Control-vs.-Suc, Control-vs.-ABA, Control-vs.-Suc + ABA). RNA sequence data analyses revealed that, when compared to the control, there were 2838 genes that were significantly differentially expressed in the Suc-treated endosperm (Fig. 1A, Supplementary Table S1). Among them, 1201 genes were up-regulated and 1637 genes were down-regulated (Fig. 1B, Supplementary Table S1). In the ABA-treated endosperm, 9396 genes were significantly differential expressed, with 3181 up-regulated and 6215 down-regulated genes (Fig. 1A,B, Supplementary Table S1). In the Suc + ABA- treated endosperm, there were 4537 significantly differentially expressed genes, including 1661 up-regulated and 2876 down-regulated genes (Fig. 1A,B, Supplementary Table S1).

The hierarchical heatmap display revealed that maize endosperm treated with a combination of exogenous Suc and ABA had a different transcript pattern from that treated with Suc or ABA alone (Fig. 1C, Supplementary Table S1). As shown in the Venn diagrams in Fig. 1A, there was an overlap of 1504 genes between Suc and ABA groups, 1426 genes between Suc and Suc + ABA, and 3111 genes between ABA and Suc + ABA (Fig. 1A). There were only 897 significantly differential genes that were commonly shared in Suc, ABA and Suc + ABA groups (Fig. 1A). In addition, there were 805, 6275 and 1494 genes that were specific to the Suc, ABA and Suc + ABA group respectively (Fig. 1A). These data suggested that the expression pattern in response to the combination of Suc and ABA was not just an additive or subtractive algorithm of those responding to Suc or ABA alone.

### Gene ontology (GO) and pathway enrichment analysis of DEGs

To further understand the function of these DEGs, gene ontology (GO) term enrichment analysis (P < 0.05) was performed (Supplementary Figure S3 and Supplementary Table S2). For DEGs in the Control-vs.-Suc + ABA comparison, the most significantly enriched GO terms were ’cellular lipid metabolic process’ (p = 6.25E-09) in the biological process (BP) group, ‘catalytic activity’ (p = 9.60E-17) in the molecular function (MF) group and ‘cytoplasmic vesicle’ (p = 2.15E-09) in the cellular component (CC) group (Supplementary Figure S3B). For DEGs in the Control-vs.-ABA comparison, the most significantly enriched GO terms were ‘nucleosome organization’ (p = 2.31E-14) in the biological process (BP) group, ‘hydrolase activity, acting on acid anhydrides’ (p = 2.54E-09) in the molecular function (MF) group and ‘chromosomal part’ (p = 1.07E-12) in the cellular component (CC) group (Supplementary Figure S3A). However, there was significantly enriched GO terms in the biological process (BP) group and only one GO terms in the molecular function (MF) ‘galactosyltransferase activity’ (p = 2E-04) and cellular component (CC) group ‘cytoplasmic vesicle’ (1.63E-05) respectively (Supplementary Figure S3C). The number of GO terms in the biological process (BP) group was more significantly in the Control-vs.-ABA comparison than in the Control-vs.-Suc + ABA comparison. However, the significantly GO terms of ‘cellular lipid metabolic process’, ‘lipid metabolic process’, ‘fatty acid metabolic process’, ‘carbohydrate metabolic process’ and ‘cellular carbohydrate metabolic process’ which were related to material metabolism in maize endosperm were enriched only in the Control-vs.-Suc + ABA comparison. This suggested that material metabolism including starch synthesis related genes might be regulated by combined action of Suc and ABA.

DEGs of the libraries Control-vs.-Suc, Control-vs.-ABA and Control-vs. -Suc + ABA were subjected to Kyoto Encyclopedia of Genes and Genomes (KEGG) pathway enrichment analysis. As a result, 1389, 4950, and 2292 DEGs were mapped to 121, 127, and 126 KEGG pathways, including biosynthesis of secondary metabolites, plant hormone signal transduction, starch and sucrose metabolism, flavonoid biosynthesis, fatty acid metabolism, and plant-pathogen interaction pathway, among others. A complete list of enriched KEGG pathways was given in Supplementary Table S3.

### Identification of candidate transcription factors induced by sucrose/ABA and involved in expression activity of endosperm starch synthesis genes

**Figure 2:**
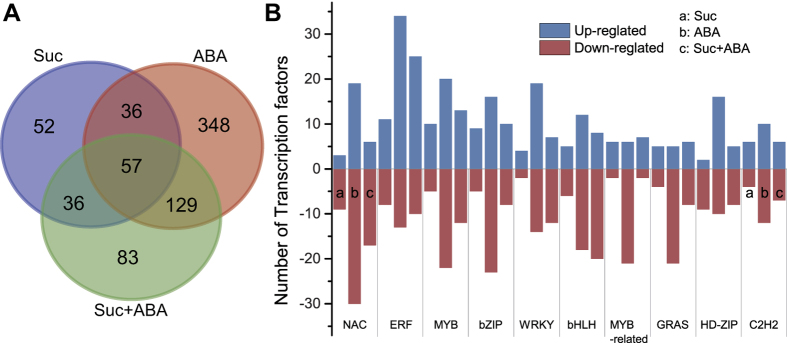
Differentially expressed transcription factor genes after treatment with sucrose, ABA, or both. Further information is presented Supplementary Table S5. (**A**) Venn diagram of the number of TFs in three libraries; (**B**) Families of differentially expressed transcription factor genes from three libraries. The x-axis indicates the distribution of different transcription factor families in the three libraries, and the y-axis represents the number of differentially expressed (up- or down-regulated) genes in each transcription factor family.

Sucrose as a signalling molecule has an important regulatory effect on the expression of genes related to starch synthesis in plants. To identify the expression of starch genes in the libraries, the reported genes expressed in maize seed (Supplementary Table S4) were searched for among the DEGs in from the three comparisons. Twenty-eight starch synthesis genes were expressed in all libraries. The DEGs showed that the expression of some starch genes increased in response to exogenous sucrose. In addition, their expression was further enhanced by co-treatment with ABA. For example, AGPS2 and PHOL were up-regulated by Suc, and SSI, SSIIIa, and bt1 were noticeably up-regulated by the combined action of Suc and ABA. We inferred from these results that sucrose and ABA also co-regulate the gene expression of starch synthesis genes in the developing endosperm of maize, which is consistent with the findings of Chen *et al*.^17^. In our RNA-seq analysis, 741 DEGs were annotated as TF genes and classified into 50 TF families according to the criteria of PlantTFDB 3.0^23^. The three pairwise comparisons (Control-vs.-Suc, Control-vs.-ABA and Control-vs.-Suc + ABA libraries) led to the identification of 181, 570, and 305 TFs, respectively (Fig. 2A and Supplementary Table S5). Fifty-seven TFs were co-up-regulated or co-down-regulated among all three libraries. Among the genes co-regulated among all three libraries, the NAC and bZIP TF family were highly induced. However, substantial numbers were regulated exclusively in Suc library (52), ABA library (348), or Suc + ABA library (83). Highly induced genes from many prominent families were found—ERF, NAC, MYB, bZIP, WRKY, bHLH, MYB-related TFs, GARS, HD-ZIP, and C_2_H_2_ (Fig. 2B). To further functional analysis of the transcription factors induced by sucrose/ABA that are involved in expression of endosperm starch synthesis genes, 47 candidate transcription factors were selected based upon their relatively high expression levels in the libraries (Supplementary Table S6). The AP2/EREBP superfamily is composed of the AP2, ERF, and RAV families. The ERF family includes the ERF and CBF/DREB subfamilies, which are involved in plant responses to abiotic stress^24^. In our study, two starch synthesis genes (GRMZM2G429899, *ZmAGPL1* and GRMZM2G141399, *ZmSSIIIa*) and a transcription factor from the AP2/EREBP superfamily (GRMZM2G421033, *ZmEREB156*) were chosen for further analysis. AGPase catalyses the first key regulatory step in the starch biosynthetic pathways present in all higher plants that produce ADP-Glc and pyrophosphate (PPi) from Glc-1-P and ATP^3^. The maize *AGPL1* gene (shrunken-2, *sh2*) encodes the large subunit of the rate-limiting starch biosynthetic enzyme, ADP-glucose pyrophosphorylase^25^. A *sh2* transgene increased maize yield by acting in maternal tissues to increase the frequency of seed development and played an important role only in the endosperm^26^,^27^. The maize *ZmSSIIIa* gene is one of the most important starch synthase genes and is mainly expressed in maize endosperm during starch biosynthesis. Mutations in maize eliminating *SSIIIa* (*du1*) produce an endosperm with a glassy, dull appearance^2^,^3^. For analysis of the cis-elements in promoters, the 2000 nucleotide sequences upstream of the transcription initiation sites of maize starch genes were used to search the PLACE (Plant Cis-acting Regulatory DNA Elements) database available online (http://www.dnaaffrc.go.jp/PLACE/signalscan). It was determined that cis-elements including the TATA-box, CAAT-box, G-box, ABRE, GARE, and Skn-1-motif (cis-acting regulatory element required for endosperm expression) were found in promoters. In addition, the cis-element SURE1 was rich in the two promoters.

### Verification of DEGs using qPCR

**Figure 3:**
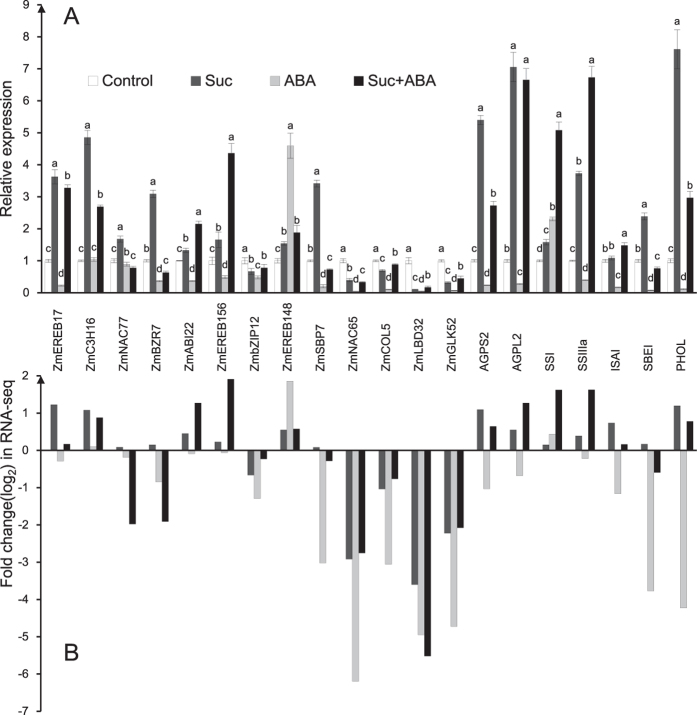
Real-time quantitative RT-PCR confirmation of 20 DEGs. (**A**) The fold change (log_2_) in gene expression based on RNA-Seq; (**B**) The relative gene expression levels determined by RT-qPCR. Relative gene expressions were normalized by comparison with the expression of 18S, and analysed using the 2^−ΔΔCT^ method. All RT-qPCRs for each gene used three biological replicates, with three technical replicates per experiment. The error bars indicate SE. Different lowercase letters indicate a significant difference, as determined by the least-significant difference test (*P* < 0.05).

To confirm the reliability of the RNA-seq results, qPCR was conducted for 20 DEGs with high or low expression levels. Additionally, qPCR was used to measure eight genes related to starch synthesis. Our RNA-seq data appeared to be reliable via RNA-seq analyses subsequently being confirmed by qPCR (Fig. 3).

### Transcriptional activation and Subcellular localization of *ZmEREB156*

**Figure 4:**
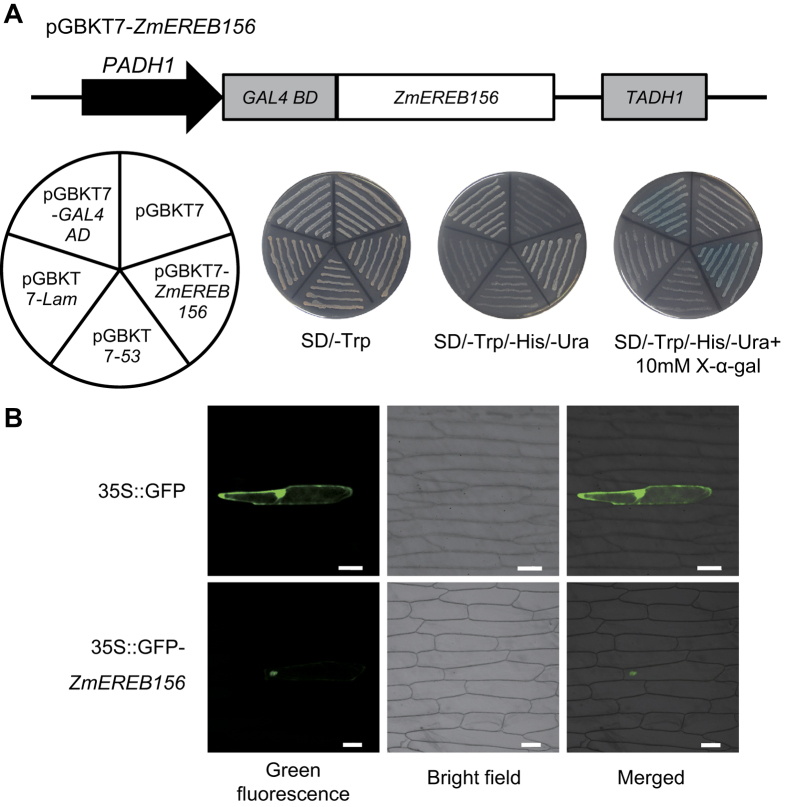
Transcriptional activation activity and subcellular localization analysis of *ZmEREB156*. (**A**) Transcriptional activation activity of the *ZmEREB156*. The structure of the pGBKT7-*ZmEREB156* plasmid; The arrangement of yeast strains on the plate; The growth of transformed yeast cells on SD/-Trp, SD/-Trp/-His/-Ura and SD/-Trp/-His/-Ura + 10 mg/mL X-α-gal medium, respectively. pGBKT7-GAL4 AD was the positive control. pGBKT7, pGBKT7-Lam, and pGBKT7-53 were negative controls. (**B**) Subcellular localization analysis of *ZmEREB156* in onion epidermal cells. Fluorescence microscopy of onion epidermal cells expressing either GFP, GFP-*ZmEREB156* as indicated (scale bar = 100 um).

To ascertain whether *ZmEREB156* has potential transcriptional activation ability, we fused the encoding region of *ZmEREB156* in frame to the GAL4 DNA binding domain in the pGBKT7 vector, and transformed the resulting construct into yeast strain AH109. The results showed that cells transformed with pGBKT7-*ZmEREB156* or pGBKT7-GAL4 AD (as a positive control) grew well on synthetic SD-Trp-His-Ura selection media and turned blue in X-α-gal indicator (Fig. 4A), demonstrating that *ZmEREB156* was a transcriptional activator in yeast.

To determine the sub-cellular localization of the *ZmEREB156* protein, the 35S:eGFP-*ZmEREB156* construct and an empty vector were introduced into onion epidermal cells via particle bombardment. GFP fluorescence was observed under a confocal microscope. Onion epidermal cells expressing the control vector showed GFP fluorescence throughout the cells (Fig. 4B). In contrast, GFP fluorescence was localized solely in the nuclei of the onion epidermal cells transformed with the 35S:eGFP-*ZmEREB156* fusion protein. These results indicated that *ZmEREB156* localized to the nucleus *in vivo*.

### Overexpression of *ZmEREB156* enhances the promoter activity of starch genes in maize endosperms

To determine whether *ZmEREB156,* in response to sucrose and/or ABA, was involved in the induction of the two starch genes (*Zmsh2* and *ZmSSIIIa*) *in vivo*, the *ZmEREB156* coding sequence was amplified by primer P156 (Supplementary Table S8) and cloned into an expression vector (pBIUbi-221) driven by the maize ubiquitin promoter (pUbi:*ZmEREB156*), which served as an effector construct (Fig. 6A). Promoters of two possible target genes were amplified from the genomic DNA of B73 by PCR using primers P1 and P2 (Supplementary Table S8) and cloned into the reporter construct (p*Promoter*:Luc).

**Figure 5:**
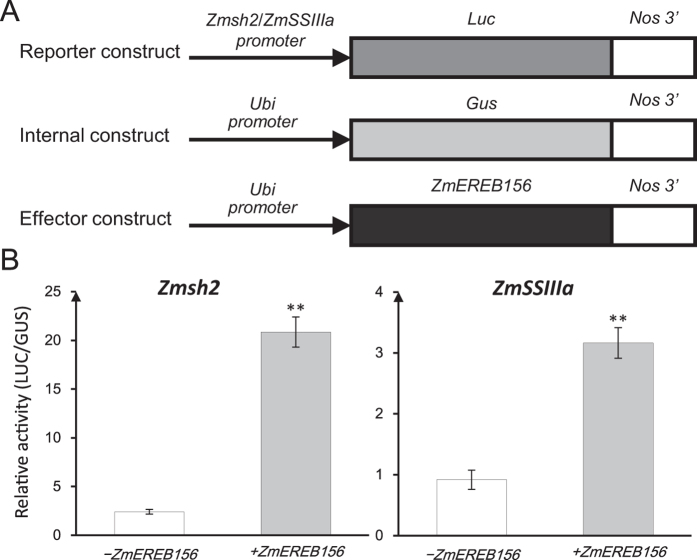
Transient assay for the interaction between *ZmEREB156* and the promoter of *Zmsh2* and *ZmSSIIIa* in maize endosperm. (**A**) Diagram of the promoters of *Zmsh2* and *ZmSSIIIa* and overexpressed-construct of *ZmEREB156*; Full-length promoters of *Zmsh2* and *ZmSSIIIa* were constructed into the report vector, and *ZmEREB156* was cloned into the effect vector, respectively; (**B**) The reporter construct, p*SSIIIa*:Luc and p*sh2*:Luc, and the internal control construct, pUbi:Gus, were co-bombarded into 10 DAP maize endosperm either with (+) or without (−) the effector construct (pUbi:*ZmEREB156*). LUC activity was normalized to GUS activity in every independent transformation. The data are given as the means ± SE of three replicates. The significance of the difference between the −*ZmEREB156* and +*ZmEREB156* conditions was analysed using a one-sided paired t-test (**P < 0.01).

The effector constructs were co-bombarded into maize endosperm with the respective reporter constructs. As shown in Fig. 5, the luciferase (LUC) activity driven by the psh2:Luc construct in the *ZmEREB156*-overexpressing endosperms was ~8.6× higher than that of endosperms not expressing *ZmEREB156*. LUC activity also increased about 3.4× in the p*SSIIIa*:Luc construct-bombarded endosperms. These findings indicate that *Zmsh2* and *ZmSSIIIa* were regulated by *ZmEREB156*, suggesting that the positive involvement of *ZmEREB156* in response to Suc may be mediated by an ABA-dependent signalling pathway.

### *ZmEREB156* binds specifically to the promoters of *ZmSSIIIa* in yeast

**Figure 6:**
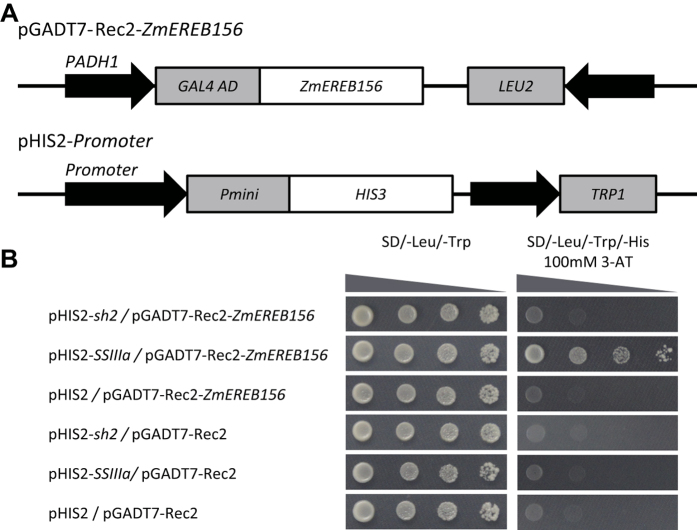
Identification of *Zmsh2* and *ZmSSIIIa* regulated by *ZmEREB156* with yeast one hybrid assay. (**A**) Schematic structure of yeast expression construct pGADT7-Rec2-*ZmEREB156* and reporter construct pHIS2-Promoter *Zmsh2* and *ZmSSIIIa* promoter); (**B**) Growth performance on SD/-Leu-/Trp and SD/-Leu-/Trp/-His medium containing 100 mM 3-AT Growth performance on SD/-Leu-/Trp and SD/-Leu-/Trp/-His medium containing 100 mM 3-AT in a series of 10-fold dilutions. pHIS2/pGADT7-Rec2-*ZmEREB156*, pHIS2-*sh2*/pGADT7-Rec2, pHIS2-*SSIIIa*/pGADT7-Rec2 and pHIS2/pGADT7-Rec2 were used as negative controls.

To confirm whether the *ZmEREB156* protein could bind to the promoter region of the two starch genes, a yeast one-hybrid assay was performed. The pGADT7-Rec2-*ZmEREB156* plasmid (containing the putative DNA-binding domain of the two transcription factors fused to the GAL4 activation domain) and the construct pHIS-cis (promoters of the two putative target genes) were co-transformed into yeast strain Y187 (Fig. 6A). As indicated by the activation of the reporter genes, *ZmEREB156* can bind to the promoters of *ZmSSIIIa* gene (Fig. 6B). These results implied that *ZmEREB156* has DNA binding activity and may directly regulate the expressions of the target gene *ZmSSIIIa*; however, *ZmEREB156* may indirectly regulate the expressions of the target gene *Zmsh2.*

## Discussion

In other plants, sucrose signals are central in determining plant growth and development by interacting with Sucrose and ABA signalling pathways^7^,^28^,^29^. However, the molecular mechanism of these interactions is complicated and still unclear. To date, no studies on the possible interactions between ABA signalling and sucrose have been reported in starch biosynthesis of maize endosperm. Sugars regulate cellular activity at multiple levels, from transcription and translation to protein stability and activity^30^. The identification and understanding of transcriptional regulatory networks is a major challenge in biology. It has been observed that sucrose affects gene expression through the regulation of other transcription factors, such as bZIP11, MYB75/PAP1 and WRKY. Increasingly, computational methods are being used that identify relationships between gene expression patterns and putative regulatory promoters or sequences in promoter regions. Information on the response of regulatory promoter or promoter elements controlling gene expression to sugar and ABA allows transcriptional networks to be understood at a molecular level.

RNA-seq analysis was used to monitor global transcriptional changes at the 10 DAP developmental stage of endosperm in B73 maize under exogenous Suc, ABA, and Suc plus ABA, which enabled comprehensive analysis of differential transcriptional genes between different treatments (Fig. 1A and Supplementary Table S1). The DEGs were mainly involved in carbohydrate metabolism, hormone signal transduction, brassinosteroid biosynthesis, and fatty acid biosynthesis pathways, which are all related to endosperm development in maize. Expression of 20 genes from the RNA-seq result was confirmed by correlation with qPCR. In addition, 47 candidate transcription factors were selected, based on their relatively high expression levels in the libraries that hold promise for future studies on molecular mechanisms underlying endosperm development in maize.

The maize endosperm treated with a combination of exogenous Suc and ABA had a different transcript pattern from that treated with Suc or ABA alone (Fig. 1C and Supplementary Table S1). Approximately 12% of differentially expressed genes responding to the combination were not found in the differentially expressed genes responding to Suc or ABA. For example, ZmEREB156 was up-regulated specifically by the combination but neither by Suc nor ABA. In contrast, ZmbHLH171 was specially down-regulated by the combination (Supplementary Table S1). In addition, there were 805, 6275 and 1494 genes that were specific to the Suc, ABA and Suc + ABA group respectively and the total number of genes exclusively up- or down- regulated by treatment of Suc or Suc + ABA is less than the number of genes exclusively regulated by ABA alone (Fig. 1A,B). These data suggested that the interaction between Suc and ABA was complicate and there might exist synergistic effect and antagonistic effect between sucrose and ABA response. Many reports have shown that sucrose up-regulates diverse genes related to the starch biosynthetic pathway, such as those that encode specific subunits of ADP-Glc pyrophosphorylase (AGPase) in different species^11^,^12^,^31^. In our study, most identified starch synthetic genes were induced by Suc or ABA, for example, AGP*S2* and *PHOL* were up-regulated by Suc, and *SSI, SSIIIa*, and *bt1* were noticeably up-regulated by the combined action of Suc and ABA (Fig. 3). Our results were consistent with Chen *et al*.^17^. These results suggest the existence of crosstalk between the sucrose and ABA signalling pathways in the starch biosynthetic pathway. Studies have suggested that sugars and ABA often have synergistic effects and antagonistic effects on diverse developmental processes in plants^32^,^33^,^34^. However, we mainly focus on the molecular mechanism of the synergistic effect between sucrose and ABA signalling involving in starch biosynthesis of maize endosperm in this paper and we will pay more attention to the antagonism effect on endosperm or seed development in maize for future research.

*ZmEREB156*, which belongs to the AP2/EREBP transcription factor family, was induced by the combined action of Suc and ABA. The AP2/EREBP gene family has divided into four subfamilies, AP2, RAV, dehydration-responsive element binding protein (DREB), and ERF (ethylene-responsive element binding factor). The RAV, DREB, and ERF subfamilies are of particular interest due to their involvement in plant responses to stress. For example, the AP2/EREBP transcription factor WRINKLED1 (*WRI1*) is involved in the regulation of seed storage metabolism in *Arabidopsis*^24^. In addition, Xiao found that *OsERF2* affected the accumulation of sucrose and UDPG by mediating expression of key genes involved in sucrose metabolism and hormone signalling pathways^35^. In our study, *Zmsh2* and *ZmSSIIIa* promoter activity increased about 8.6 times and 3.4 times, respectively, after overexpression of *ZmEREB156*. Interestingly, *ZmEREB156* could bind to the promoters of *ZmSSIIIa* and but not bind to the promoter of *Zmsh2* (Figs 5 and 6). These findings indicate that *ZmSSIIIa* was under the regulation of *ZmEREB156*, suggesting that the positive involvement of *ZmEREB156* in the responses to Suc may be mediated by an ABA-dependent signalling pathway and the interaction between Suc and ABA was synergistic effect. It also suggests that *ZmEREB156* might indirectly regulate the expressions of the target gene *Zmsh2*, and *Zmsh2* might be co-regulated by *ZmEREB156* with other transcription factors co-mediated by Suc/ABA signalling. A similar conclusion was reported by Grimault^36^. For example *WRI1*, which encodes the main regulator of lipid biosynthesis in the seed, is a direct target of *LEC2*. However, *LEC2* also acts directly on genes involved in reserve accumulation including *OLE*1, encoding an oleosin, and *At2S1–S4* and 2S-like, which encode seed storage proteins. Direct targeting by *LEC2* is mediated by its B3 domain, which binds specifically to RY-motifs such as CATGCA. It was also deduced that *ZmEREB156* plays a distinctive role in regulating the co-expression of several starch synthesis genes in maize endosperm through sucrose and ABA signalling. To date, demonstration of protein–protein interactions between starch biosynthetic enzymes has been confined to endosperm of cereal species. Some rice starch biosynthetic isozymes are physically associated with each other and form active protein complexes^37^. Abe showed that there is a close interaction among *SSI, BEI*, and *BEIIb* during amylopectin biosynthesis in rice endosperm^38^. This means that the requirement for multiple, synergistic signals of Suc and/or ABA is probably correlated with the complexity of the mechanism underlying starch synthesis pathway in maize endosperm. Further investigation is needed into the integral regulatory network of *ZmEREB156* at the molecular level and the functional linkage of related maize AP2/EREBP factors that co-regulate starch synthesis in maize endosperm.

Production of stable, fertile transgenic lines is an essential technique in gene function studies; however, transformation in maize is difficult, with low transformation rates and long breeding times. Therefore, using transient transformation approaches to study gene function is an alternative method^39^,^40^. Because the *ZmEREB156* gene was expressed in maize endosperm, 10 DAP endosperm was used as the receptor of transient expression, which was conducted in order to determine the regulatory effect of the *ZmEREB156* protein on the expression of the starch biosynthesis gene. Transient expression in maize endosperms is a good method for identifying the roles of target genes expressed in endosperm and has been used successfully in our previous studies^41^,^42^,^43^. In conclusion, we speculate that the *ZmEREB156* gene might play a role in the starch biosynthesis via the synergistic effect of sucrose and ABA based on a series of experimental validation.

## Materials and Methods

### Plant materials and growth conditions

*Zea mays* L. B73 inbred seedlings were grown in the field under recommended agronomic guidelines and self-pollinated. When silks emerged, strict self-pollinations were performed between 11:00 and 13:00 every day.

Ten DAP (days after pollination), maize endosperms from three independent plants were immersed in 1/2 MS liquid culture with the plant hormone and sugar treatments added [Control: 200 mM mannitol (added to the samples without sugars as an osmotic control); Suc: 200 mM sucrose, ABA: 200 mM mannitol supplemented with 100 μM abscisic acid; and Suc + ABA: a mixture of 200 mM Suc and 100 μM ABA] and shaken (80 rpm) at 28 °C in the dark for 24 h^44^. The individual plants were composited by treatment, and four cDNA libraries were constructed based on total RNAs extracted from the maize endosperms to use in RNA sequencing (RNA-seq) analysis. The optimum concentration of each treatment was defined as that at which the gene demonstrated a response to exogenous sucrose and ABA^44^. The timing of maize endosperm collection was based on previous reports that the developmental steps associated with endosperm function, including the activation of storage product synthesis and deposition, initiate at 8 to 10 DAP^45^,^46^,^47^,^48^. Furthermore, genes associated with energy reserve and carbohydrate biosynthesis are up-regulated at 10 DAP according to Transcriptome Sequencing^49^. We therefore selected 10 DAP maize endosperms for our experiments.

### RNA extraction and library preparation for RNA-seq

Total RNA was extracted using TRIzol reagent (Invitrogen) and purified using the RNA easy Mini RNA kit (Qiagen). RNA quality and quantity were verified using a Nano Drop 1000 spectrophotometer and an Agilent 2100 Bioanalyzer prior to further processing at the Beijing Genomics Institute (BGI; Shenzhen, China). On-column DNase digestion was performed according to the manufacturer’s protocol. The total RNA was treated with DNase I prior to library construction, and poly-(A) mRNA was purified with Magnetic Oligo d (T) beads (Dynabeads). The mRNA was fragmented by treatment with divalent cations and heat. First-strand cDNA was transcribed from the cleaved RNA fragments using reverse transcriptase and random hexamer primers; second-strand cDNA synthesis followed, using DNA polymerase I and RNaseH. The double-stranded cDNA was subjected to end-repair using T4 DNA polymerase, the Klenow fragment, and T4 polynucleotide kinase. This was followed by a single <A> base addition using Klenow 3′ to 5′ exo-polymerase and ligation to an adaptor or index adaptor using T4 DNA ligase. Adaptor-ligated fragments were separated by size on an agarose gel, and cDNA fragments of the desired range (200 ± 25 bp) were excised from the gel. PCR was performed to selectively enrich and amplify the cDNA fragments. After validation with an Agilent 2100 Bioanalyzer and ABI StepOnePlus Real-Time PCR System, the cDNA library was sequenced on a flow cell using the Illumina HiSeq^TM^ 2000 sequencing platform. The sequence data were deposited in the NCBI Sequence Read Archive (http://www.ncbi.nlm.nih.gov/Traces/sra) under accession number SRP068962.

### Statistical and functional analyses of DEGs

Clean reads were obtained after filtering the adaptor, empty, low-quality, and one-copy tags from raw reads. Next, they were aligned to the maize reference gene set based on B73 genome (release 5b.60) by applying the SOAPaligner/SOAP2 programs^22^. The calculation of gene expression used the reads per kb per millon reads (RPKM) method^50^. DEGs (FDR < 0.01, |log2 Ratio| ≥ 1) were analysed by comparing the Suc, ABA, and Suc + ABA libraries with the control library (Control-vs.-Suc, Control-vs.-ABA, Control-vs.-Suc + ABA). To analysis the gene expression pattern under the treatment of Suc or/and ABA, we firstly selected all of differentially expressed genes from the three pairwise comparisons (|log2 (fold change)| ≤6) and then employed the cluster software for a hierarchical heatmap^51^ (http://bonsai.hgc.jp/~mdehoon/software/cluster/). DEGs were annotated and classified into TF families according to the criteria of PlantTFDB 3.0^49^. TFs were identified from the three pairwise comparisons (Control-vs.-Suc, Control-vs.-ABA and Control-vs.-Suc + ABA libraries) and selected according to their relatively high expression levels in libraries. The highly expressed TFs were subjected to confirmative analysis involving starch biosynthesis in maize endosperm.

### Gene ontology (GO) and pathway enrichment analysis of DEGs

DEGs were subjected to GO functional enrichment and KEGG pathway analysis. Gene ontology annotation was carried out using Blast2GO software (v.2.5.0)^52^. GO enrichment analysis used hypergeometric test to find significantly enriched GO terms in the input list of DEGs, based on GO::TermFinder^53^,^54^. KAAS (KEGG Automatic Annotation Server) provides functional annotation of genes by BLAST comparisons against the manually curated KEGG genes database. The result contains KO (KEGG Orthology) assignments and automatically generated KEGG pathways.

### Verification of changes in gene expression using qPCR

To confirm the reliability of the RNA-seq results, qPCR was conducted using the iQ5 real-time PCR detection system (Bio-Rad, CA, USA) and SYBR Green PCR Master Mix (Takara, Dalian, China). Twenty-three DEGs were tested using primer sequences listed in Supplementary Table S7. Sample collection and treatment were as described under “Plant materials and growth conditions”, this paper.

Total RNA was extracted using TRIzol (Invitrogen, CA, USA) and treated with DNase I (TaKaRa) to remove any genomic DNA. Reverse transcription-PCR (RT-PCR) was performed with 1 μg of total RNA using the PrimeScript™ RT reagent kit (TaKaRa). The specificity of qPCR primers was confirmed by melting curve analyses. The Ct value was determined using the instrument’s software. The relative quantification of gene expression was monitored after normalization to 18S rRNA expression as an internal control. The relative transcription levels were calculated using the 2^–ΔΔCt^ method. The qPCR results were obtained from three biological replicates and three technical replicates for each gene and sample.

### Subcellular localization of transcription factors

The complete coding sequence of *ZmEREB156*, minus the stop codon, was ligated into the pCAMBIA2300-35S-eGFP vector after amplification by PCR using primer G156 (Supplementary Table S8) with *Bam*HI and *Xba*I restriction sites to create the fusion construct (pCAMBIA2300-*ZmEREB156*-eGFP). The empty pCAMBIA2300-35S-eGFP vector was used as a control. The fusion construct and the control were introduced into onion (*Allium cepa* L.) epidermal cells by particle bombardment using PDS-1000/He (Bio-Rad)^50^. The transformed cells were incubated on 1/2 MS medium for 24–48 h at 28 °C. The subcellular localization of GFP fusion proteins was visualized with a fluorescence microscope BX61 (Olympus, Japan).

### Analysis of transcriptional activation in yeast cells

A transcription activation assay was performed in yeast strain AH109 according to the Yeast Protocols Handbook (Clontech). The full-length coding region of *ZmEREB156* was generated by PCR using primer BD156 (Supplementary Table S8). The PCR product was cloned into the pGBKT7 vector using *Eco*RI and *Bam*HI sites and was named pGBKT7–*ZmEREB156*. Plasmids pGBKT7, pGBKT7-Lam, and pGBKT7-53 were used as negative controls. The pGBKT7-GAL4 AD was used as a positive control. These constructs were transformed into yeast strain AH109 by the lithium acetate method. After confirmation by screening on selective medium plates without tryptophan (SD/-Trp), the positive colonies were transferred onto SD/-Trp/-His/-Ura selective medium with or without X-α-D-Galactosidase (X-α-gal), and the yeast cells were photographed after incubating the plates at 30 °C for 3–4 d to evaluate transcription activity.

### Particle bombardment and transient expression assays

To investigate whether the candidate transcription factors induced by sucrose and/or ABA regulated the expression activity of endosperm starch synthesis genes, transient expression assays were performed. The *ZmEREB156* coding sequence, spanning from ATG to the stop codon, was amplified by primer P156 (Supplementary Table S8) and cloned into an expression vector (pBIUbi-221) driven by the maize ubiquitin promoter (pUbi:*ZmEREB156*), which served as an effector construct (Fig. 5A). The upstream ~2000 bp promoters of two possible target genes (*Zmsh2* and *ZmSSIIIa*) were amplified from the genomic DNA of B73 by PCR using primers P1 and P2 (Supplementary Table S8) and cloned into the reporter construct p*Promoter*:Luc (p*Zmsh2*:Luc and p*ZmSSIIIa*:Luc) (Fig. 5A). The pUbi:Gus was used as an internal construct (Fig. 5A). For the analysis of the reporter constructs, the molar ratio of the reporter construct to the internal construct plasmid DNA (pUbi:Gus) was 2:1. To analyse the effector constructs’ effect on promoter expression, the reporter construct, effector construct, and internal construct were combined at a molar ratio of 2:2:1 and then co-bombarded into maize endosperm using a PDS-1000/He (Bio-Rad)^50^. Each independent experiment consisted of three replicates and was repeated 3–4 times with similar results. After particle bombardment, the medium plates containing bombarded endosperms were sealed and incubated at 28 °C in the dark for 24 h. The fluorescence and luminescence were determined using a Luminoskan Ascent (Thermo, IL, USA)^50^.

### Promoter binding in yeast one-hybrid system

A yeast one-hybrid assay was used to investigate whether *ZmEREB156* binds to the promoters of the two starch genes. The promoter sequences (Zm*sh2* and *ZmSSIIIa*) were generated by PCR using primers H1 and H2 (Supplementary Table S8) and cloned into the yeast expression vector pHIS2 between the *Eco*RI/*Sma*I and *Eco*RI/*Sac*I sites. The *ZmEREB156* coding region was amplified by primer R156 (Supplementary Table S8) and cloned into the pGADT7-Rec2 vector using *Eco*RI/*Xho*I sites to obtain pGADT7-Rec2-*ZmEREB156*. The yeast strain Y187 was co-transformed with pGADT7-Rec2-TFs and pHIS2 vectors; pHIS2/pGADT7-Rec2-*ZmEREB156,* pHIS2/pGADT7-Rec2, pHIS2-*SSsh2*/pGADT7-Rec2, and pHIS2-*SSIIIa*/pGADT7-Rec2 were co-transformed as negative controls. The DNA-protein interactions were evaluated according to the growth status of yeast cells cultured on SD/-His/-Leu/-Trp selective medium with 100 mM 3-amino-1,2,4-triazole (3-AT) for 3 d.

## Additional Information

**How to cite this article**: Huang, H. *et al*. Sucrose and ABA regulate starch biosynthesis in maize through a novel transcription factor, *ZmEREB156. Sci. Rep.* **6**, 27590; doi: 10.1038/srep27590 (2016).

## Supplementary Material

Supplementary Information

Supplementary Table S1

Supplementary Table S2

Supplementary Table S3

Supplementary Table S5

## Figures and Tables

**Table 1:** Summary of read numbers based on the RNA-seq data from 10 DAP maize endosperm under treatment with sucrose and ABA.

**Map to Gene**	**Control**	**ABA**	**Suc**	**Suc + ABA**
Total reads	52,802,328	54,469,252	52,802,328	54,469,252
Total base pairs	4,752,209,520	4,902,232,680	4,752,209,520	4,902,232,680
Total mapped reads	40,845,708	44,414,887	41,222,336	41,128,823
Perfect match	32,907,332	35,290,266	32,654,624	32,715,975
<=5 bp Mismatch	7,938,376	9,124,621	8,567,712	8,412,848
Unique match	39,194,164	42,663,993	39,502,779	39,401,228
Multi-position match	1,651,544	1,750,894	1,719,557	1,727,595
Total unmapped reads	11,956,620	10,054,365	11,579,992	13,340,429
