# Supplementary material for: Sucrose and ABA regulate starch biosynthesis in maize through a novel transcription factor, ZmEREB156
Source: Sci Rep. 2016 Jun 10;6:27590. doi: 10.1038/srep27590 (PMC4901336; doi:10.1038/srep27590)
Supplement: Supplementary Information [file srep27590-s1.pdf]

Sucrose and ABA regulate starch biosynthesis in maize through a novel transcription factor, *ZmEREB156*

Huanhuan Huang[1], Sidi Xie[1], Qianlin Xiao[1], Bin Wei[1], Lanjie Zheng[1], Yongbin Wang[1], Yao Cao[1], Xiangge Zhang[1], Tiandan Long[1], Yangping Li[1], Yufeng Hu[1], Guowu Yu[1], Hanmei Liu[2], Yinghong Liu[3], Zhi Huang[4], Junjie Zhang[2]*, and Yubi Huang[1]*

[1] College of Agronomy, Sichuan Agricultural University, Chengdu 611130, Sichuan, China

[2] College of Life Science, Sichuan Agricultural University, Chengdu 611130, Sichuan, China

[3] Maize Research Institute, Sichuan Agricultural University, Chengdu 611130, Sichuan, China

[4] College of Horticulture, Sichuan Agricultural University, Chengdu 611130, Sichuan, China

***To whom correspondence should be addressed.**

Email: yubihuang@sohu.com; junjiezh@163.com

List of Supplementary Information

Table S1. List of differential expression genes ($P \leq 0.05$ and |log2 Ratio| ≥1) during different three treatments (Suc, ABA and Suc+ABA) identified by comparing with control treatment.

Table S2. GO enrichment analysis of DEGs among three comparison.

Table S3. KEEG pathway enrichment analysis of DEGs among three comparison.

Table S4. Starch synthesis genes in maize reported previously.

Table S5. Transcription factor genes were identified respectively from the DEGs of three comparisons group according to the annotation of PlantTFDB 3.0.

Table S6. List of 47 candidate transcription factors.

Table S7. Primers of 20 genes analyzed using qRT-PCR.

Table S8. Primers used for vector construction in paper.

Figure S1. Distributions of reads on reference genes of four RNA-seq libraries.

Figure S2. Distribution of genes' coverage in four RNA-seq libraries.

Figure S3. GO annotation clusters of differentially expressed genes.

Table S4. Starch synthesis genes in maize reported previously.

Alternative Name	Locus	Chr	Gene Length(bp)	Protein Length(aa)	Key reference
AGPS1a(bt2)	GRMZM2G068506	4	1754	476	Hannah,L.C. et al., 2001
AGPS1b	GRMZM2G163437	1	1614	517	Hannah,L.C. et al., 2001
AGPS2	GRMZM2G106213	2	1927	508	Lai,J. et al.,2004
AGPL1(sh2)	GRMZM2G429899	3	1911	470	Giroux,M.J., 1992
AGPL2	GRMZM2G027955	6	2123	321	Giroux,M., 1994
AGPL3	GRMZM2G144002	7	1995	514	Jiang,H. et al., 1995
AGPL4	GRMZM2G391936	1	1832	515	Jiang,H. et al., 2007
GBSSI(wx)	GRMZM2G024993	9	1818	605	Weil,C.F. et al., 1992
GBSSIIa	GRMZM2G008263	7	1905	609	Liu,F. et al., 2012
SSI	GRMZM2G129451	9	2357	640	Knight,M.E. et al., 1998
SSIIa(su2)	GRMZM2G348551	6	2865	731	Harn,C. et al., 1998
SSIIb-2	GRMZM2G105791	5	2569	704	Schnable,P.S. et al., 2009
SSIIc	GRMZM2G126988	5	2943	775	Yan,H. et al., 2009
SSIIIa(du1)	GRMZM2G141399	10	6027	1674	Gao,M. et al., 1998
SSIIIb-1	GRMZM2G121612	10	3944	1191	Jiang,H. et al., 2007
SSIV	GRMZM2G044744	8	3316	909	Jiang,H. et al., 2008
SSV	GRMZM2G130043	4	2613	701	Schnable,P.S. et al., 2009
ISA1(su1)	GRMZM2G138060	4	2712	818	James,M.G. et al., 1995
ISO2	GRMZM2G090905	6	2829	799	Dinges,J.R. et al., 2003
ISO3	GRMZM2G150796	7	2793	694	Dinges,J.R. et al., 2003
zpu1	GRMZM2G158043	2	3261	962	Beatty,M.K. et al., 1999
SBEI	GRMZM2G088753	5	2797	823	Baba,T. et al.,1991
SBEIIa	GRMZM2G073054	2	2795	881	Gao,M. et al., 1997

Table S6. List of 47 candidate transcription factors.

TF-ID	Name	TF-ID	Name	TF-ID	Name
GRMZM2G021777	ZmCOL3	GRMZM2G024530	ZmbHLH171	GRMZM2G023872	ZmGRAS20
GRMZM2G421033	ZmEREB156	GRMZM2G144275	ZmbHLH136	GRMZM2G129777	ZmEREB192
GRMZM2G135052	ZmMYBR69	GRMZM2G080583	ZmTHX17	GRMZM2G029323	ZmEREB17
GRMZM2G137802	ZmWRKY68	GRMZM2G016434	ZmEREB129	GRMZM5G813892	ZmMYBR43
GRMZM2G008356	ZmABI22	GRMZM2G083504	ZmbHLH121	GRMZM2G071034	ZmC3H16
GRMZM2G024973	ZmGRAS44	GRMZM2G128807	ZmbHLH127	GRMZM2G145444	ZmMYB50
GRMZM2G398506	ZmWRKY1	GRMZM2G035103	ZmC2H2	GRMZM2G168079	ZmbZIP75
GRMZM2G310368	ZmEREB147	GRMZM2G160838	ZmMYB32	AC196475.3	ZmNAC77
GRMZM2G109869	ZmGRAS52	GRMZM2G159399	ZmARF17	AC198937.4	ZmNAC37
GRMZM2G336533	ZmNAC60	GRMZM2G088443	ZmbHLH104	AC194970.5	ZmBZR7
GRMZM2G363052	ZmEREB94	GRMZM2G161315	ZmZHD2	GRMZM2G124524	ZmEREB85
GRMZM2G320827	ZmTHX32	GRMZM2G371033	ZmSBP18	GRMZM2G369799	ZmMYB127
GRMZM2G123119	ZmEREB177	GRMZM2G405699	ZmABI49	GRMZM2G078820	ZmMYB158
GRMZM2G027960	ZmC2H2	GRMZM2G466044	ZmEREB195	GRMZM2G180328	ZmNAC20
GRMZM2G421212	ZmGATA19	GRMZM2G033356	ZmbHLH130	GRMZM2G023872	ZmGRAS20
GRMZM2G059939	ZmEREB162	GRMZM5G878561	ZmSBP22		

Table S7. Primers of 20 genes analyzed using qRT-PCR.

Name	Genes ID	Forward primer	Reverse primer
ZmEREB17	GRMZM2G029323	ACCCTCCTCCTACACCACCT	GCGTAAGGCGACGAGTAGAG
ZmC3H16	GRMZM2G071034	CGCTCATAGCGAAAGAGAGC	CAGCTGGTTGCATAGGTTCA
ZmNAC77	AC196475.3_FG004	AAGTGGATCCTCCACGACTG	GACGATGACCTTGCCATTTT
ZmBZR7	AC194970.5_FG001	ACAAAACGACGTACCCGAAG	CCTTCTCCTTGTCGAAGCAA
ZmABI22	GRMZM2G008356	GCGGGAGATGCTATTCTCAG	CAGCCACAATGTACCCTCCT
ZmEREB156	GRMZM2G421033	CATCTGGCTGGGGTCCTAC	ATCTCCTCGGGGAAGTTGAG
ZmbZIP12	GRMZM2G448607	GTCCTCGAGGTGCTCCAGAT	GGGTCGTCGGTGACCATC
ZmEREB148	GRMZM2G053503	AGGTGGAGGCACAGACTCAG	GATGCCGAGGAAGTTCATGT
ZmSBP7	GRMZM2G098557	ATGCTGGCTGAGATTCACCT	AATTGTCGGGGCAAATTACA
ZmNAC65	GRMZM2G043813	GGTCTTCGGTCACTTGTTGC	GAAGCTGCACCCGTAGTCTC
ZmCOL5	GRMZM2G075562	GCATCAGTCCGATGTCTTGA	GGCTTGCCTTTTATGTCCAG
ZmLBD32	GRMZM2G121487	CGGTCTCTGCAGGTATCACA	ATAGGGCGCAAATATGCAGT
ZmGLK52	GRMZM2G124495	TGGCCTCACAATCTACCACA	GCAGCAGCTCGTTTCTCTTT
AGPS3	GRMZM2G106213	TATTGGGCCTTGATCCTGAA	TCTCTGAGAAGCCGAAGCAT
AGPL2	GRMZM2G027955	CGATCCGAAAACACCATTCT	AAGAAGCAGCCGTGTGAAAT
SSI	GRMZM2G129451	CTGGAGTGGGTATTCCCTGA	TCACGATTCGATCTGCTGTC
SSIIIa	GRMZM2G141399	GATGCGTTGTAGCAAGTCCA	AGTCTTGGCGCATAGTTGCT
ISAI	GRMZM2G138060	CCAAATTCCGCAAAGAATGT	CTCTGACCAGTCAGGCTTCC
SBEI	GRMZM2G088753	TAGCAGCAGATCAGGCACAC	TGCATGGCTATGGACAACAT
PHOL	GRMZM2G074158	AAGTTCTCCGCTTGAAGCAA	CCTCCCAGTTGAGAGACTCG
18S	EU957665	AGCTAAAGTTGTCTCGCCCT	CGTTCGCCACTTTGTTCTCA

Table S8. Primers used for vector construction in paper.

	Forward primer	Reverse primer
G156	ggatccATGATGCAGGGCGAGTACCG	tctagaCTCGGCGTCGGAGCTCTC
BD156	gaattcATGATGCAGGGCGAGTACCG	ggatccTCACTCGGCGTCGGAGCT
P1	ctgcagAATATAGTACCGGGACAGTCTTTC	ggatccTATCTCCTCCAACTACTAGATACAC
P2	gcatgcCAAGAAACACGACATCCTATCTC	ggatccCTAGAAGGGGAAGAAAAGAAGG
P156	ggatccATGATGCAGGGCGAGTACCG	acgcgtTCACTCGGCGTCGGAGCT
H1	gaattcAATATAGTACCGGGACAGTCTTTC	cccgggTATCTCCTCCAACTACTAGATACAC
H2	gaattcCAAGAAACACGACATCCTATCTC	gagctcCTAGAAGGGGAAGAAAAGAAGG
R156	gaattcATGATGCAGGGCGAGTACCG	ctcgagTCACTCGGCGTCGGAGCT

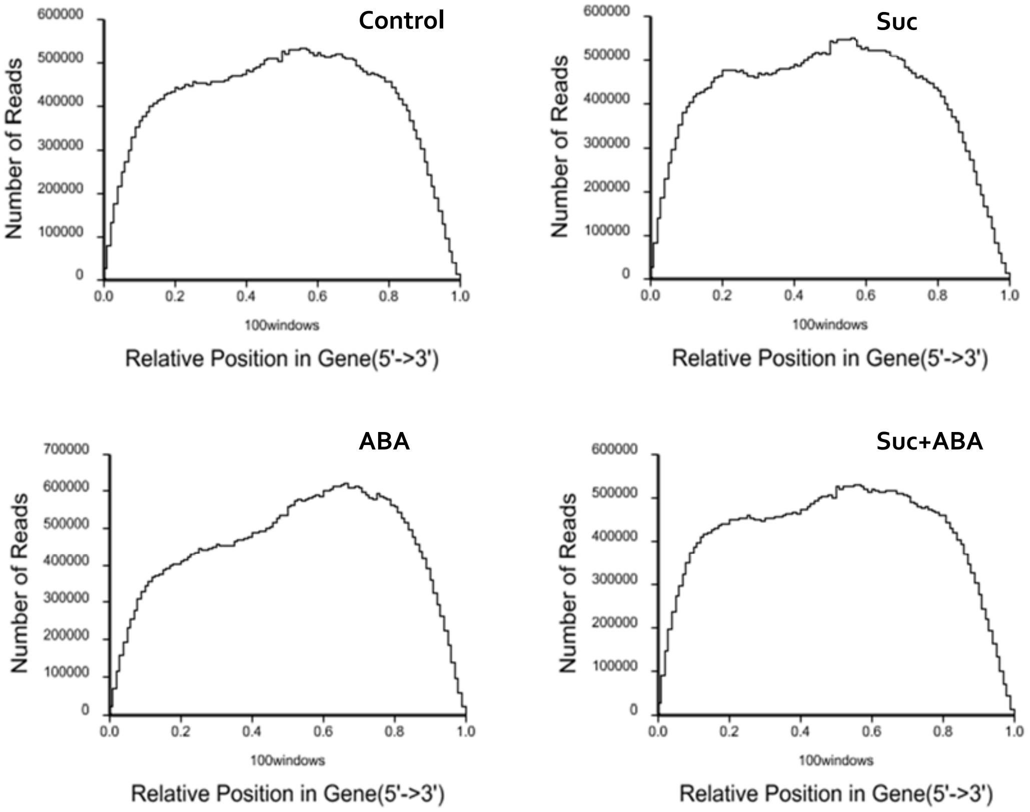

Supplementary Fig. S1 Distributions of reads on reference genes of four samples. Tens of thousands of genes of different lengths may be present in a sample. The locus positions in those genes were normalized by dividing the gene bodies into 100 windows, then counting reads mapped to every window and using this value to calculate the read distribution along genes.

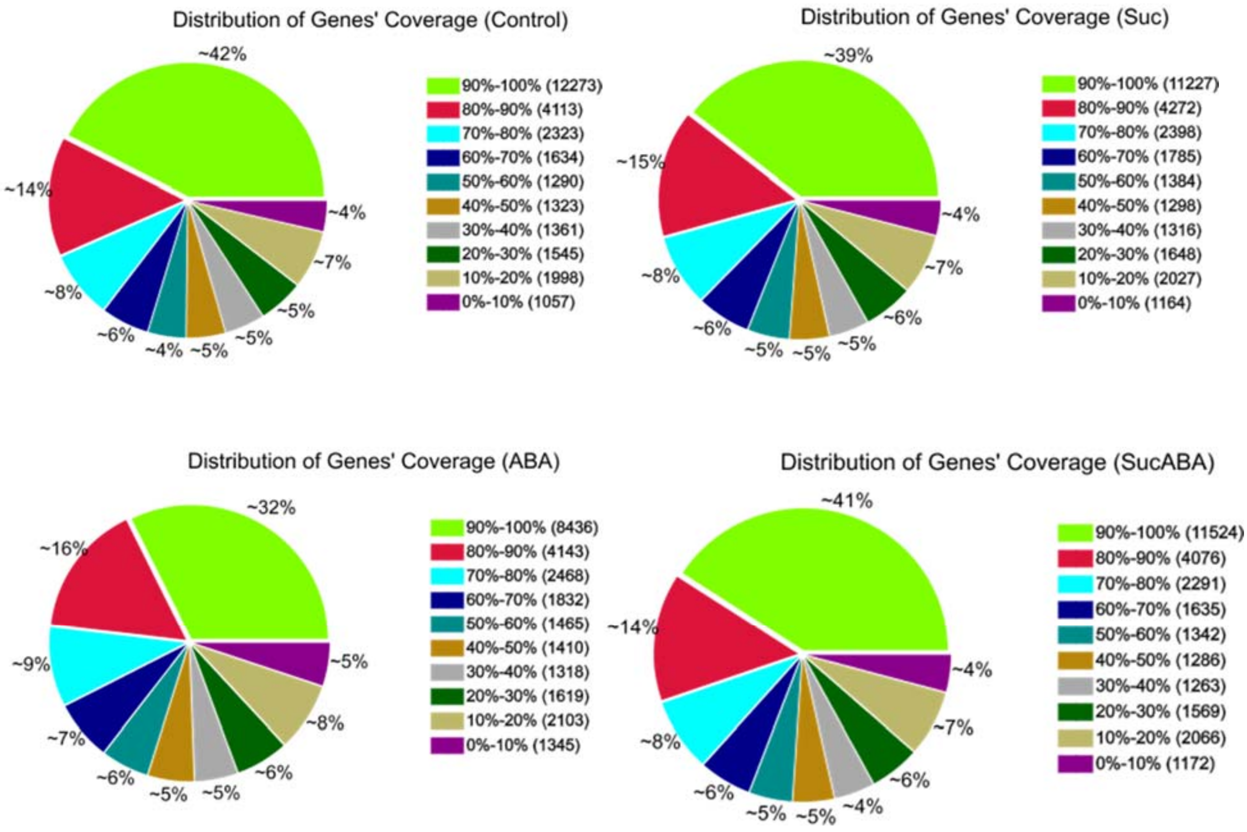

Supplementary Fig. S2 Distribution of gene coverage in four RNA-seq libraries. Gene coverage is calculated as the percentage of a gene covered by reads. This value is the ratio of the base number in a gene covered by unique mapping reads to the total base number of the coding region for that gene.

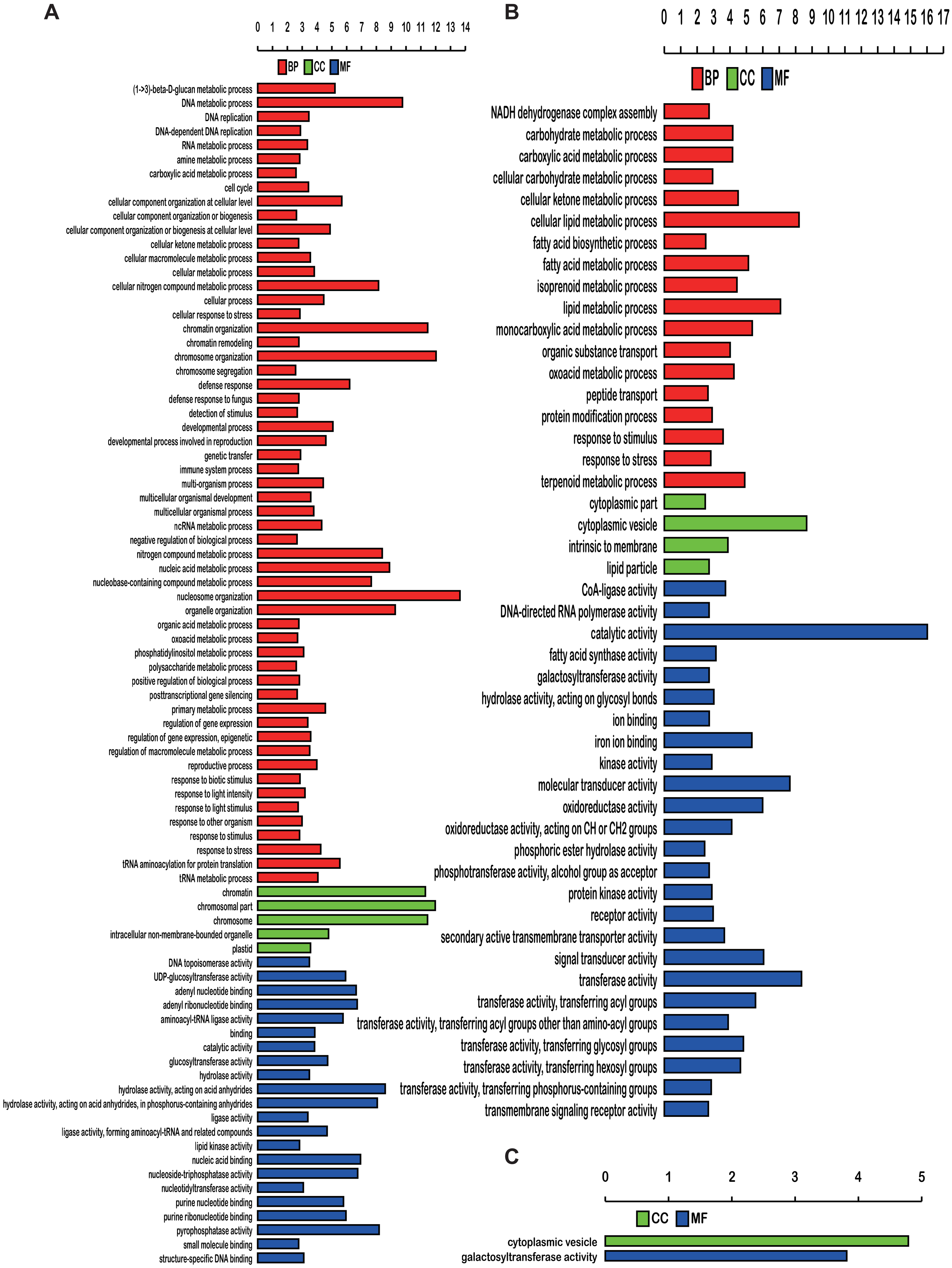

Supplementary Fig.S3 Significantly enriched Gene Ontology (GO) terms (P <0.05) in the differentially expressed genes in the three comparison libraries. GO terms belong to biological processes (BP), molecular functions (MF), and cellular components (CC) were shown in red, green, blue, and green, respectively. X-axis represented enriched GO term and Y-axis meant the –log10 (P-value). (A) GO terms in the Control vs. ABA comparison; (B) GO terms in the Control vs. SucABA comparison; (C) GO terms in the Control vs. Suc comparison
